# Supplementary material for: Surface Properties of Synaptosomes in the Presence of L-Glutamic and Kainic Acids: In Vitro Alteration of the ATPase and Acetylcholinesterase Activities
Source: Membranes (Basel). 2021 Dec 17;11(12):987. doi: 10.3390/membranes11120987 (PMC8708669; doi:10.3390/membranes11120987)
Supplement: Supplementary file 1 [file membranes-11-00987-s001.zip › membranes-1483104-supplementary.pdf]

Supplementary Material

Surface properties of synaptosomes in the presence of L-glutamic and kainic acids: *in vitro* alteration of the ATPase and acetylcholinesterase activities

Virjinia Doltchinkova[1,*], Nevena Mouleshkova[1], and Victoria Vitkova[2]

[1] Faculty of Biology, Sofia University "St. Kliment Ohridski", 8 Dragan Tsankov blvd., 1164 Sofia, Bulgaria
[2] Institute of Solid State Physics, Bulgarian Academy of Sciences, 72 Tzarigradsko Chaussee Blvd., 1784 Sofia, Bulgaria
[*] Corresponding author's e-mail: dolchinkova@biofac.uni-sofia.bg

Electroformation of giant unilamellar vesicles on ITO-coated electrodes from POPC in 50 mM Tris-HCl buffer in the presence of L-glutamic or kainic acids

A series of experiments is conducted to find the most appropriate protocol producing a high yield of quasispherical GUVs with diameters of the order of tens of micrometers in the aqueous media studied. Experimental protocols previously established for the electroformation of giant unilamellar vesicles (GUVs) [1, 2] are applied with modifications. Electroformation is performed in a chamber consisting of two indium tin oxide (ITO)-coated glass plates separated by a 3 mm-thick polydimethylsiloxane (PDMS, Dow Corning, Midland, MI, USA) spacer [2]. Lipid depots are uniformly spread from 50 μL of the lipid solution (1 g/L in $CHCl_3$:CH_3OH 9:1 v/v) on the conductive side of each ITO-electrode. Solvents are completely evaporated under vacuum and the chamber is filled with bidistilled water (Figure S1) or the relevant aqueous solution (Figures S2, S3, S4 and S5), and then tightly closed to avoid evaporation during electroformation. AC voltage with frequency depending on the aqueous medium, in which the electroformation is performed (see figure legends below), is applied to the chamber with peak-to-peak amplitude U_{pp} successively increased by 0.5 V every 5 minutes to 4 V. The result from the electroformation is monitored under an inverted microscope Axiovert 100 (Zeiss, Germany) equipped with a dry long-working distance objective (LD-Achroplan Ph2, 63×, NA 0.75). The figure legends report the voltage and kinetic parameters for the corresponding aqueous milieu.

In buffer solutions containing the above mentioned concentrations of L-glutamic or kainic acids the amount of quasispherical fluctuating vesicles satisfying all criteria for goodness of the experimental method [3] is reduced compared to water (Figure S1). The hydration of POPC depots by 50 mM Tris-HCl, pH 7.2 with higher ionic strength ($I = 0.050\ M^{-1}$) requires the application of modified electroformation protocols at higher frequency (Figure S2). The presence of 10 mM kainic acid in the buffer (Figure S3) leads to the production of predominantly tense, non-fluctuating vesicles, which are not applicable to bending elasticity measurements by flicker spectroscopy. In 10 mM L-Glutamic acid (Figure S4) we obtain foam-like membrane structures and aggregates of adhered vesicles remaining stable after detachment from lipid depots. These vesicular formations are inappropriate for shape fluctuation analysis. Ten-fold reduction of glutamate concentration in the buffer results in a high yield of well-fluctuating vesicles without observable membrane defects (Figure S5), suitable for measurement of membrane bending modulus.

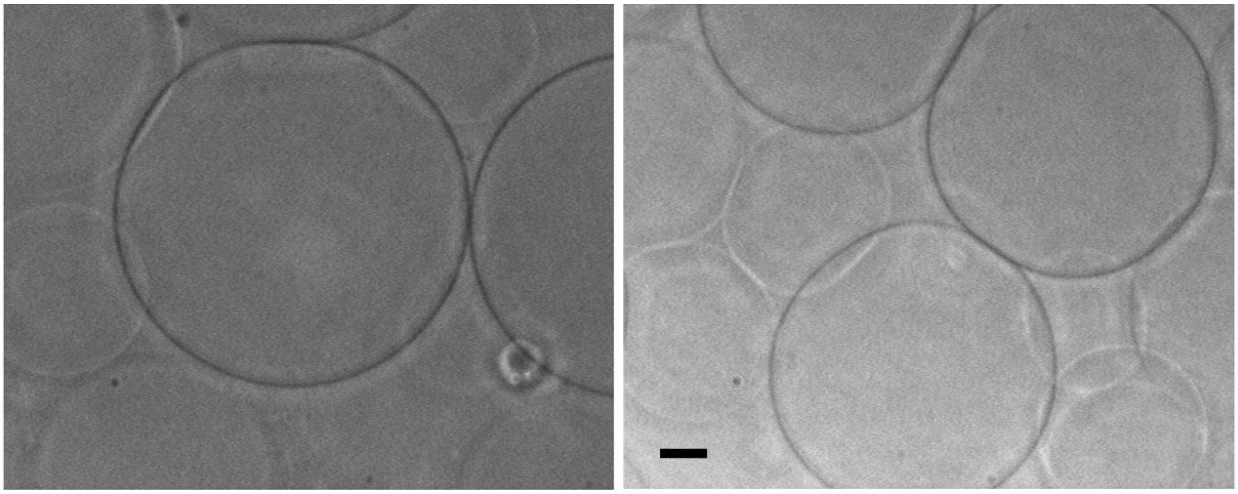

(a) (b)

Figure S1. Phase-contrast snapshots of POPC electroformation in bidistilled water at U_{pp}= 4 V (peak-to-peak), 10 Hz for (a) 3 hours; (b) 6 hours. The scale bar corresponds to 10 μm.

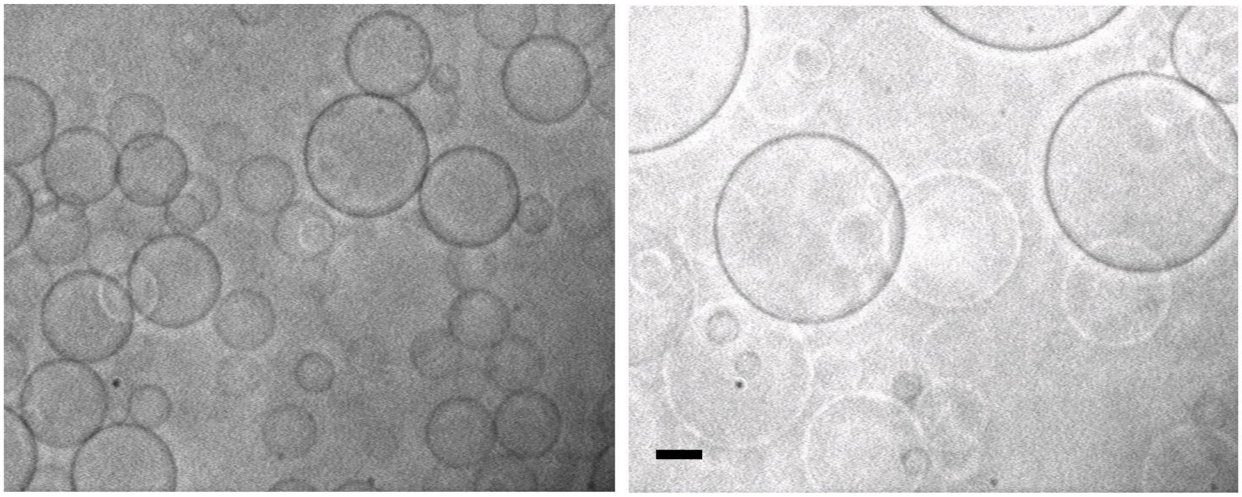

(a) (b)

Figure S2. Phase-contrast snapshots of vesicle preparations from POPC in 50 mM Tris-HCl, pH 7.2 via electroformation at U_{pp}= 4 V (peak-to-peak), 500 Hz, for (a) 4 hours; (b)8 hours. The scale bar corresponds to 10 μm.

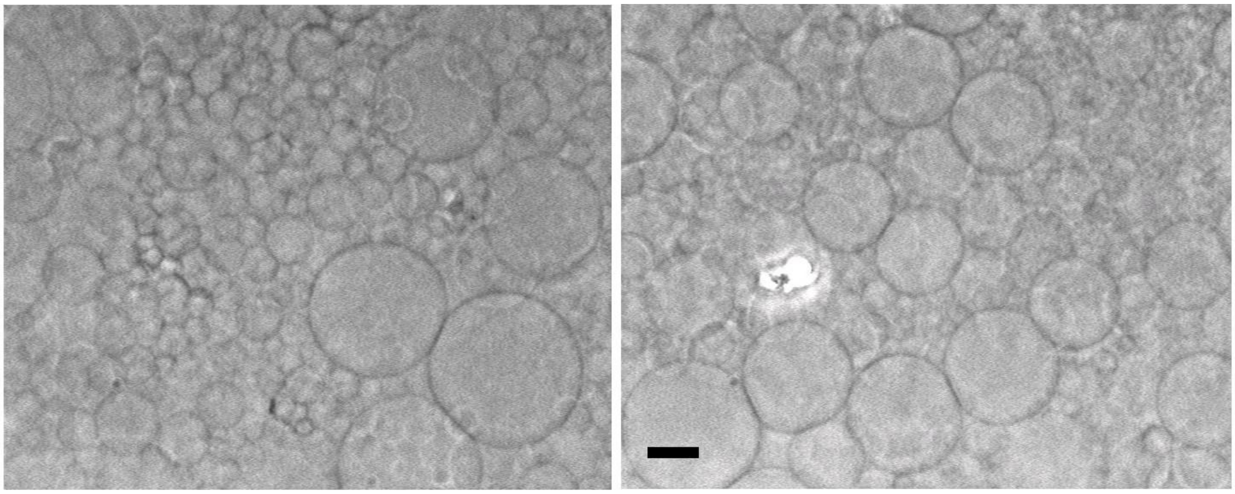

(a) (b)

Figure S3. Phase-contrast snapshots of vesicle preparations from POPC in 10 mM kainic acid, 50 mM Tris-HCl, pH 7.2 via electroformation at U_{pp}= 4 V (peak-to-peak), 500 Hz for (a) 6 hours; (b) 12 hours. The scale bar corresponds to 10 μm.

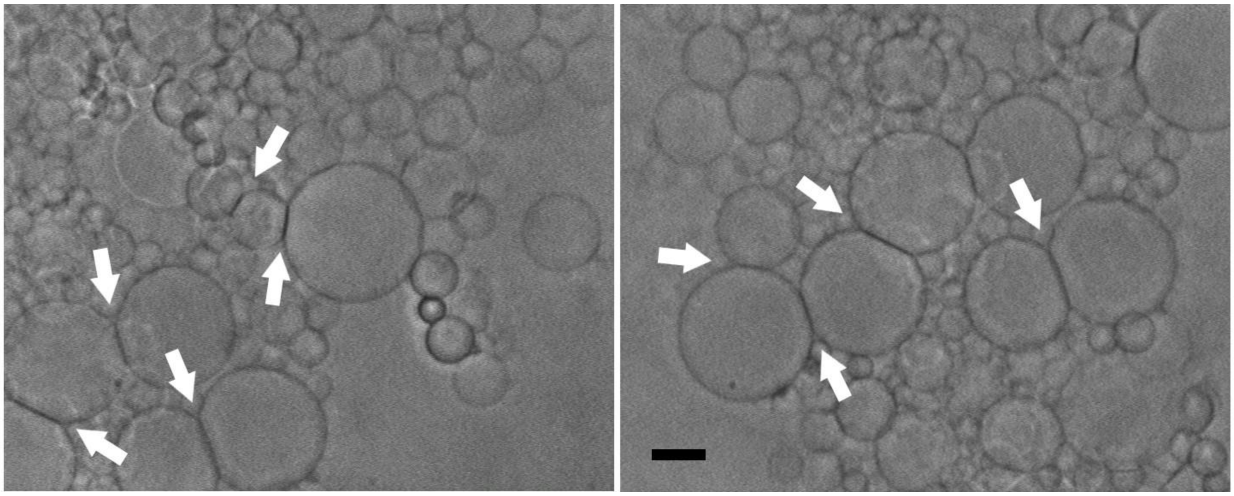

(a) (b)

Figure S4. Phase-contrast snapshots of vesicle preparations from POPC in 10 mM L-glutamic acid, 50 mM Tris-HCl, pH 7.2; electroformation at U_{pp}= 4 V (peak-to-peak) and 10 Hz for (a) 12 hours; (b) for 24 hours. White arrows point at contact patches of adjacent vesicles adhered during electroformation. These aggregates remain stable after detachment of vesicles from lipid depots. The scale bar corresponds to 10 μm.

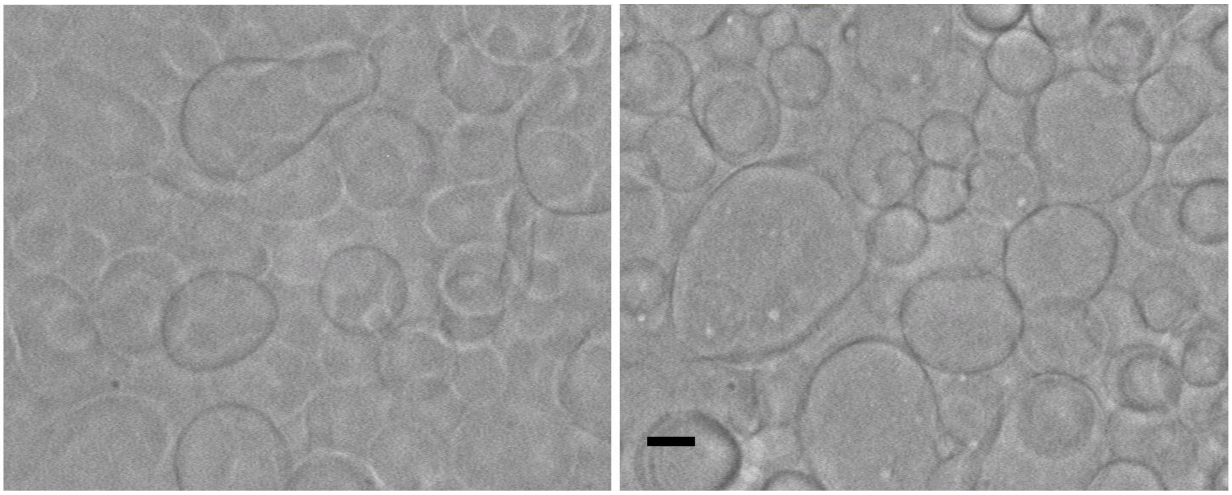

(a) (b)

Figure S5: Phase-contrast snapshots of vesicle preparations from POPC in 1 mM L-glutamic acid, 50 mM Tris-HCl, pH 7.2 via electroformation at U_{pp}= 4 V (peak-to-peak), 500 Hz for (a) 6 hours; (b) 8 hours. The scale bar corresponds to 10 μm.

Experimental data for the membrane bending elasticity of POPC GUVs

in the presence of neurotransmitters

Measurements of the bending elasticity are performed by thermal shape fluctuation analysis according to [3]. Flaccid quasispherical vesicles with diameters of the order of 10 μm and larger are chosen for thermal shape fluctuation analysis (TSFA). Several hundred images are acquired once per second. The membrane bending constant and tension of every studied vesicle are obtained by TSFA. For the calculation of the bending modulus only vesicles satisfying all selection criteria for quality (for details see [3, 4]) are considered. In Table S1 we present TSFA results for the radiusR_{ves}, bending constant k_c, and reduced membrane tension $\bar{\sigma} = \sigma R^2/k_c$ of all recorded and analyzed GUVs [3, 5, 6].

Table S1. TSFA results for the radius R_{ves}, membrane bending modulusk_c, and tension $\bar{\sigma}$ of POPC GUVs; GF – goodness of fit (χ^2-test).

R_{ves}, μm	k_c, 10^{-19}J	$\bar{\sigma}$	GF
(*i*) *Control, H_2O, pH 5.6*			
9.26	1.91±0.53	34±14	0.39
14.99	1.78±1.45	59±52	0.68
9.06	5.21±3.09	40±31	0.33
5.89	1.04±0.23	10±6	0.49
5.14	0.94±0.24	9±5	0.54
4.81	0.96±0.26	24±13	0.32
6.12	1.52±0.21	-1.1±0.8	0.35
6.89	1.49±0.33	4.1±3.1	0.44
12.01	3.12±1.67	95±62	0.40
17.93	5.97±4.88	102±95	0.33
8.91	1.48±0.26	9.2±5.5	0.43
8.87	1.35±0.51	14±10	0.57

6.83	1.91±0.79	40±22	0.31
9.76	1.05±0.68	29±14	0.40
13.48	1.80±0.71	21±15	0.63
6.88	2.30±1.37	30±26	0.29
5.90	1.20±0.27	31±13	0.61
(*ii*) *Control, 0.05 M Tris-HCl, pH 7.22*			
5.62	1.86±0.97	22±17	0.54
8.10	1.02±0.18	16±7	0.56
6.58	1.19±0.15	-1.2±0.9	0.58
9.03	3.11±0.99	26±12	0.46
5.66	1.28±0.25	5.3±3.0	0.45
6.64	1.76±0.71	53±29	0.58
7.56	1.09±0.37	29±17	0.39
17.87	2.14±0.64	52±22	0.44
1 mM L-Glutamic acid, 0.05 M Tris-HCl, pH 7.22			
5.72	1.06±0.69	17±15	0.48
6.16	1.12±0.23	7.4±4.8	0.57
7.12	2.09±0.91	26±17	0.50
5.87	0.84±0.22	4.5±3.6	0.44
5.89	1.06±0.33	6.7±5.2	0.25
12.13	1.22±0.19	18±5	0.38
6.55	0.90±0.11	-1±0.9	0.47
4.80	0.88±0.22	2.7±1.8	0.59
10.02	0.83±0.09	-1.1±0.9	0.46
10 mM Kainic acid, 0.05 M Tris-HCl, pH 7.22			
4.87	0.97±0.44	14±9	0.46
9.54	1.59±0.64	21±13	0.51
7.17	2.31±1.45	42±28	0.56
6.21	1.48±0.21	8±6	0.23
11.48	1.30±0.11	22±10	0.61

References:

[1] Angelova, M. I.; Dimitrov, D. S. Liposome electroformation. *Faraday Discuss. Chem. Soc.* **1986**, *81*, 303-311, https://doi.org/10.1039/DC9868100303.

[2] Vitkova, V.; Antonova, K.; Popkirov, G.; Mitov, M. D.; Ermakov, Y. A.; Bivas, I. Electrical resistivity of the liquid phase of vesicular suspensions prepared by different methods. *J. Phys.: Conf. Ser.* **2010**, *253*, 012059.

[3] Genova, J.; Vitkova, V.; Bivas, I. Registration and analysis of the shape fluctuations of nearly spherical lipid vesicles. *Phys. Rev. E Stat Nonlin. Soft Matter Phys.* **2013**, *88* (2), 022707, doi:10.1103/PhysRevE.88.022707.

[4] Vitkova, V.; Misbah, C. Dynamics of lipid vesicles – from thermal fluctuations to rheology. In *Advances in Planar Lipid Bilayers and Liposomes*, Iglič, A., Ed.; Academic Press, Burlington, 2011; pp. 258-292.

[5] Faucon, J.F.; Mitov, M.D.; Méléard, P.; Bivas, I.; Bothorel, P. Bending elasticity and thermal fluctuations of lipid membranes. Theoretical and experimental requirements. *J. Physique* **1989**, *50* (17), 2389-2414, doi:10.1051/jphys.0198900500170238900. jpa-00211069.

[6] Mitov, M. D.; Faucon, J. F.; Méléard, P.; Bothorel, P. Thermal fluctuations of membranes, In *Advances in Supramolecular Chemistry*, Gokel, G.W., Ed.; JAI Press Inc., Greenwich, CT, 1992; pp. 93-139.
